# Tris[4-(dimethyl­amino)­pyridinium][(bis-μ-dichlorido)-deca­aqua­dichlorido­dineodymium(III)] penta­chloride dihydrate

**DOI:** 10.1107/S1600536812041724

**Published:** 2012-10-10

**Authors:** Meriem Benslimane, Hocine Merazig, Jean-Claude Daran, Ouahida Zeghouan

**Affiliations:** a Unité de Recherche de Chimie de l’Environnement et Moléculaire Structurale, Faculté des Sciences Exactes, Département de Chimie, Université Mentouri de Constantine, 25000 Constantine, Algeria; b Laboratoire de Chimie de Coordination, UPR-CNRS 8241, 205 route de Narbonne, 31077 Toulouse Cedex 4, France

## Abstract

The title compound, (C_7_H_11_N_2_)_3_[Nd_2_Cl_4_(H_2_O)_10_]Cl_5_·2H_2_O, consists of three 4-(dimethyl­amino)­pyridinium cations, one of which is disordered about an inversion center, one [Nd_2_Cl_4_(H_2_O)_10_]^2+^ dication possessing inversion symmetry, five chloride anions, one of which is disordered over two inversion centers, and two lattice water mol­ecules. The 4-(dimethyl­amino)­pyridinium cations are protonated at the pyridine N atoms and form N—H⋯Cl hydrogen bonds with Cl^−^ counter-ions. The dimethyl­amino groups (C/N/C) lie close to the plane of the pyridinium rings, making dihedral angles of 1.6 (6)° and 6.5 (3)°. In the crystal, the [Nd_2_Cl_4_(H_2_O)_10_]^2+^ dications are linked *via* O—H⋯O and O—H⋯Cl hydrogen bonds, forming sheets lying parallel to the *bc* plane. These sheets are linked *via* O—H⋯Cl hydrogen bonds, forming a three-dimensional network. The 4-(dimethyl­amino)­pyridinium cations are located in the cavities and linked to the framework by C—H⋯Cl interactions.

## Related literature

For the crystal structures of complexes involving 4-(dimethyl­amino)­pyridinium, see: Chao *et al.* (1977 ▶); Mayr-Stein & Bolte (2000 ▶); Lo & Ng (2008 ▶, 2009 ▶); Koon *et al.* (2009 ▶); Benslimane *et al.* (2012 ▶). For hydrogen-bond motifs, see: Bernstein *et al.* (1995 ▶).
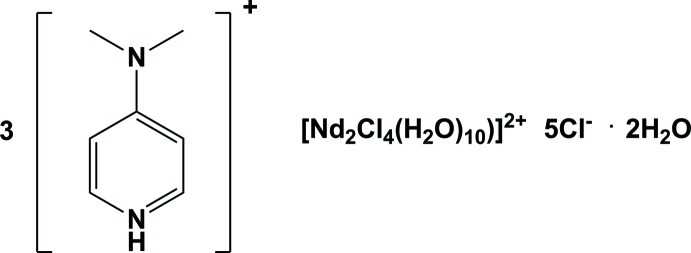


## Experimental

### 

#### Crystal data

(C_7_H_11_N_2_)_3_[Nd_2_Cl_4_(H_2_O)_10_]Cl_5_·2H_2_O
*M* *_r_* = 1193.26
Triclinic, 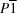

*a* = 9.5172 (4) Å
*b* = 10.7739 (5) Å
*c* = 11.9976 (5) Å
α = 74.855 (4)°
β = 69.780 (4)°
γ = 85.075 (4)°
*V* = 1114.28 (8) Å^3^
*Z* = 1
Mo *K*α radiation
μ = 2.90 mm^−1^
*T* = 180 K
0.36 × 0.22 × 0.16 mm

#### Data collection

Agilent Xcalibur Sapphire1 diffractometer
Absorption correction: multi-scan (*CrysAlis PRO*; Agilent, 2011 ▶) *T*_min_ = 0.475, *T*_max_ = 0.633
23060 measured reflections
4551 independent reflections
4088 reflections with *I* > 2σ(*I*)
*R*_int_ = 0.034

#### Refinement

*R*[*F* ^2^ > 2σ(*F* ^2^)] = 0.029
*wR*(*F* ^2^) = 0.068
*S* = 1.11
4551 reflections
210 parameters
H-atom parameters constrained
Δρ_max_ = 0.95 e Å^−3^
Δρ_min_ = −1.76 e Å^−3^

### 

Data collection: *CrysAlis PRO* (Agilent, 2011 ▶); cell refinement: *CrysAlis PRO*; data reduction: *CrysAlis PRO*; program(s) used to solve structure: *SIR92* (Altomare *et al.*, 1993 ▶); program(s) used to refine structure: *SHELXL97* (Sheldrick, 2008 ▶); molecular graphics: *ORTEPIII* (Burnett & Johnson, 1996 ▶) and *ORTEP-3 for Windows* (Farrugia, 2012 ▶); software used to prepare material for publication: *SHELXL97*.

## Supplementary Material

Crystal structure: contains datablock(s) I, global. DOI: 10.1107/S1600536812041724/su2506sup1.cif

Structure factors: contains datablock(s) I. DOI: 10.1107/S1600536812041724/su2506Isup2.hkl

Additional supplementary materials:  crystallographic information; 3D view; checkCIF report

## Figures and Tables

**Table 1:** Hydrogen-bond geometry (Å, °)

*D*—H⋯*A*	*D*—H	H⋯*A*	*D*⋯*A*	*D*—H⋯*A*
O11—H111⋯Cl4^i^	0.85	2.16	3.010 (3)	177
O11—H112⋯Cl11^ii^	0.85	2.30	3.149 (4)	173
O12—H121⋯Cl6^iii^	0.85	2.24	3.080 (3)	168
O12—H122⋯Cl3^iv^	0.85	2.25	3.089 (3)	171
O13—H131⋯O11	0.85	2.19	2.738 (6)	122
O13—H131⋯Cl4^i^	0.85	2.99	3.631 (6)	134
O13—H132⋯Cl4	0.85	2.16	3.005 (4)	172
O14—H142⋯Cl4^iv^	0.85	2.46	3.159 (4)	140
O14—H142⋯Cl11	0.85	2.73	3.210 (4)	118
O15—H151⋯O1*W*	0.85	1.84	2.683 (4)	172
O15—H152⋯Cl3	0.85	2.26	3.109 (3)	175
N12—H12⋯Cl11^v^	0.88	2.65	3.358 (5)	138
N12—H12⋯Cl11	0.88	2.78	3.451 (5)	134
O1*W*—H11*W*⋯Cl3^vi^	0.85	2.35	3.197 (3)	179
O1*W*—H12*W*⋯Cl6	0.85	2.52	3.349 (3)	167
N32—H32*A*⋯Cl4	0.88	2.26	3.0808 (16)	156
C34—H34⋯Cl1^i^	0.95	2.82	3.703 (3)	155

Symmetry codes: (i) 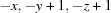; (ii) 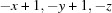; (iii) 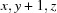; (iv) 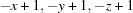; (v) 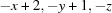; (vi) 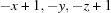.

**Fig. 1.**
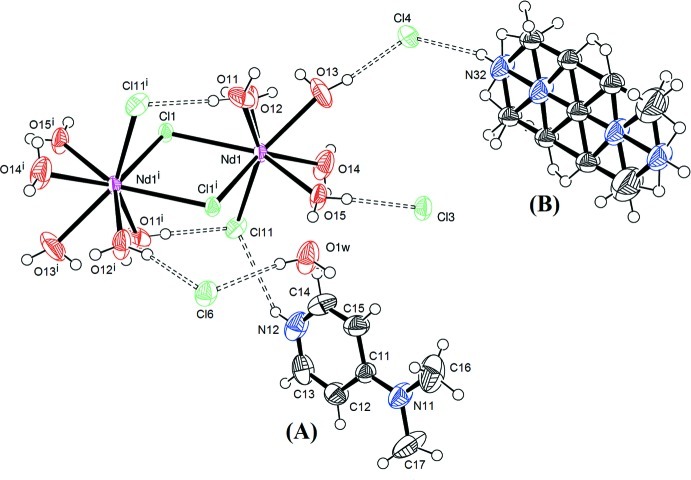
The molecular structure of the title compound, showing the atom-numbering scheme. Displacement ellipsoids are drawn at the 50% probability level. O-H···Cl and N-H···Cl hydrogen bonds are shown as double dashed lines [symmetry code: (i) -x+1, -y+1, -z]. The 4-(dimethylamino)pyridinium cations consists of ordered (A) and disordered (B) entities. The organic entity (B) is disordered about an inversion center.

**Fig. 2.**
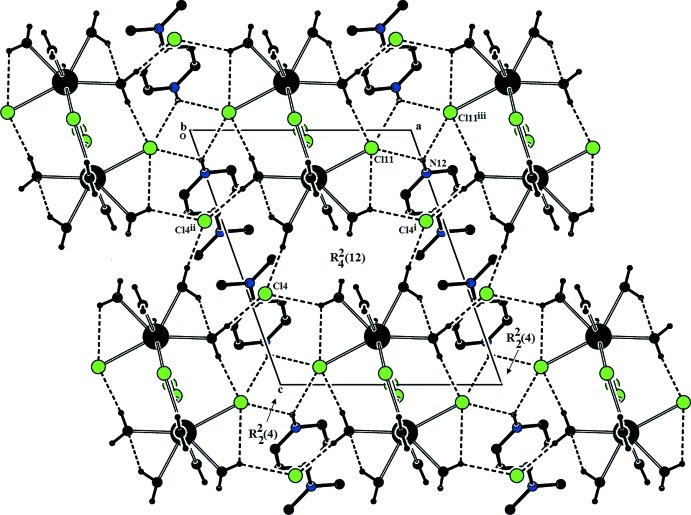
A view along the b axis of the hydrogen-bonded network of the title compound, showing the formation of ring motifs via O-H···Cl and N-H···Cl hydrogen-bonds (dashed lines) [symmetry codes: (i) -x+1, -y+1, 1-z); (ii) -x, -y+1, -z+1; (iii) -x+2,-y+1, -z].

**Fig. 3.**
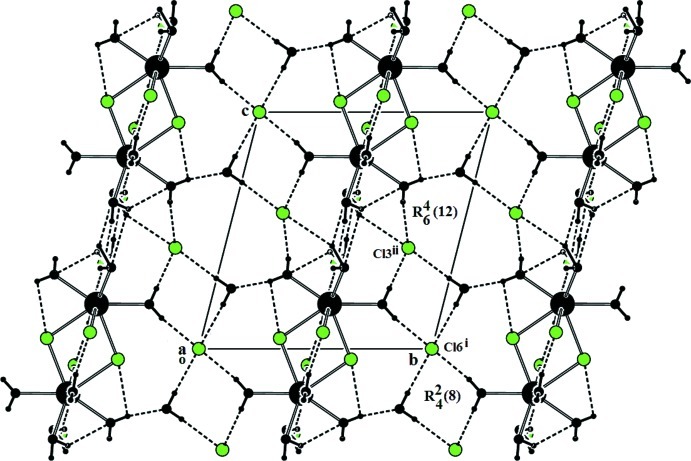
A view along the a axis of the hydrogen-bonded network of the title compound, showing the formation of ring motifs via O-H···Cl hydrogen-bonds (dashed lines) [symmetry codes: (i) x, y+1, z); (ii) -x+1, -y+1, -z+1].

## supplementary crystallographic information

### Comment

We report herein on the synthesis and crystal structure of a new hybrid organic-inorganic compound. It consists of alternating organic-inorganic layers characterized by isolated anions, as found with other compounds involving 4-(dimethylamino)pyridinium (Chao *et al.*, 1977; Mayr-Stein & Bolte, 2000; Lo and Ng, 2008, 2009; Koon *et al.*, 2009).

The asymmetric unit of the title compound contains one half of a centrosymmetric [Nd_2_Cl_4_(H_2_O)_10_]^2+^ dication, and one and one half 4-(dimethylamino)pyridinium cations, two and one half chloride anions, and one water molecule (Fig. 1). The organic cations consist of ordered (A) and disordered (B) entities (Fig. 1). They are protonated on the nitrogen atoms N12 and N32 of the pyridine rings. The organic entity (B) is disordered about an inversion center.

The two Nd atoms of the dication are linked by two bridging chloride atoms with an Nd1···Nd1^i^ separation of 4.5158 (4)Å and an Nd1-Cl1-Nd1^i^ angle of 105.74 (3)° (symmetry code: (i) -x+1, -y+1, -z) . Each Nd^III^ atom is coordinated by the O atoms of five water molecules with the Nd-O distances ranging from 2.404 (3) to 2.479 (4) Å, and by three chloride ions with bond Nd1-Cl11 being 2.7851 (10) while the bridging Nd1-Cl1 and Nd1-Cl1^i^ distances are 2.8298 (8) and 2.8345 (9) Å, respectively.

In the 4-(dimethylamino)pyridinium cations, the N-C bonds linking the dimethylamino substituent to the pyridinium ring are short [1.330 (5) and 1.2855 (12) Å], suggesting some delocalization in the cation. The dimethylamino groups (C16/N11/C17 and C36/N32/C37) lie close to the plane of their respective pyridinium rings, with dihedral angles of 1.6 (6) ° and 6.5 (3) °, respectively.

In the crystal, each Cl^-^ anion accepts hydrogen bonds which can be divided into two groups. The first group involves hydrogen bonds linking atom Cl11 with two organic cations via the pyridinium N12-H12 H atom (Table 1), generating centrosymmetric R^2^_2_(4) motifs (Bernstein *et al.*, 1995) at y = 1/2, and this arrangement is analogous to that seen in (C_7_H_10_N_2_)_2[_LaCl(H_2_O)_8]_Cl_4_^.^3H_2_O (Benslimane *et al.*, 2012). The second type of hydrogen bond, in which the Cl^-^ anion is the acceptor, is a linkage between the (free and coordinated) water molecules and the Cl^-^ anion which enlcose R^2^_4_(12) ring motifs along direction [001] (Fig. 2 and Table. 1). The [Nd_2_Cl_4_(H_2_O)_10_]^2+^ dications are linked via O-H···O and O-H···Cl hydrogen bonds to form sheets lying parallel to the bc plane (Fig. 3). These sheets are linked via O-H···Cl hydrogen bonds to form a three dimentional framework. The 4-(dimethylamino)pyridinium cations are located in the cavities. The dimeric cations are linked *via* hydrogen bonds between Cl^-^ anions and water molecules resulting in the formation of centrosymmetric R^2^_4_(8) and R^4^_6_(12) rings along direction [001] (Fig. 3 and Table. 1).

### Experimental

4-(Dimethylamino)pyridine (1 mmol, 0.08g for) and hydrochloric acid (1M) was added slowly to an aqueous solution of NdCl_3_.6H_2_O (1mmol, 0.08g). The mixture was refluxed at 353 K for about 1 h and then cooled to room temperature. Slow evaporation of the solvent at room temperature lead to the formation of colourless plate-like crystals of the title compound.

### Refinement

The 4-(dimethylamino)pyridinium cation (B) was found to be disordered about an inversion center. The disorder was treated using the PART -1 instruction in SHELXL97 (Sheldrick, 2008.) Restraints using the SAME instruction were applied to maintain a reasonable geometry. The ADP's were also restrained to have similar values. The occupancy factors were fixed at 0.5. The H-atoms of the coordinated water molecules were located in difference Fourier syntheses and were initially refined using distance restraints (O-H = 0.85 (2) Å, and H···H = 1.40 (2) Å, with *U*_iso_(H) = 1.5*U*_eq_(O). In the last cycles of refinement, they were constrained to ride on their parent O atoms. The N-bound and C-bound H atoms were included in calculated positions and refined using a riding model: N-H = 0.88 Å, C-H = 0.95 (aromatic), 0.98 (methyl) Å, with *U*_iso_(H) = 1.5*U*_eq_(C) for the methyl groups and 1.2*U*_eq_(C) for other H atoms.

### Crystal data

**Table:**

(C_7_H_11_N_2_)_3_[Nd_2_Cl_4_(H_2_O)_10_]Cl_5_·2H_2_O	*Z* = 1
*M**_r_* = 1193.26	*F*(000) = 594
Triclinic, *P*1	*D*_x_ = 1.778 Mg m^−^^3^
Hall symbol: -P 1	Mo *K*α radiation, λ = 0.71073 Å
*a* = 9.5172 (4) Å	Cell parameters from 15563 reflections
*b* = 10.7739 (5) Å	θ = 3.0–28.5°
*c* = 11.9976 (5) Å	µ = 2.90 mm^−^^1^
α = 74.855 (4)°	*T* = 180 K
β = 69.780 (4)°	Platelet, colourless
γ = 85.075 (4)°	0.36 × 0.22 × 0.16 mm
*V* = 1114.28 (8) Å^3^	

### Data collection

**Table:**

Agilent Xcalibur Sapphire1 diffractometer	4551 independent reflections
Radiation source: fine-focus sealed tube	4088 reflections with *I* > 2σ(*I*)
Graphite monochromator	*R*_int_ = 0.034
Detector resolution: 8.2632 pixels mm^-1^	θ_max_ = 26.4°, θ_min_ = 3.0°
ω scans	*h* = −11→11
Absorption correction: multi-scan (*CrysAlis PRO*; Agilent, 2011)	*k* = −13→13
*T*_min_ = 0.475, *T*_max_ = 0.633	*l* = −14→14
23060 measured reflections	

### Refinement

**Table:**

Refinement on *F*^2^	Primary atom site location: structure-invariant direct methods
Least-squares matrix: full	Secondary atom site location: difference Fourier map
*R*[*F*^2^ > 2σ(*F*^2^)] = 0.029	Hydrogen site location: inferred from neighbouring sites
*wR*(*F*^2^) = 0.068	H-atom parameters constrained
*S* = 1.11	*w* = 1/[σ^2^(*F*_o_^2^) + (0.0222*P*)^2^ + 2.5244*P*] where *P* = (*F*_o_^2^ + 2*F*_c_^2^)/3
4551 reflections	(Δ/σ)_max_ < 0.001
210 parameters	Δρ_max_ = 0.95 e Å^−^^3^
0 restraints	Δρ_min_ = −1.76 e Å^−^^3^

### Special details

**Table:**

Geometry. Bond distances, angles etc. have been calculated using the rounded fractional coordinates. All su's are estimated from the variances of the (full) variance-covariance matrix. The cell esds are taken into account in the estimation of distances, angles and torsion angles
Refinement. Refinement of F^2^ against ALL reflections. The weighted R-factor wR and goodness of fit S are based on F^2^, conventional R-factors R are based on F, with F set to zero for negative F^2^. The threshold expression of F^2^ > 2sigma(F^2^) is used only for calculating R-factors(gt) etc. and is not relevant to the choice of reflections for refinement. R-factors based on F^2^ are statistically about twice as large as those based on F, and R- factors based on ALL data will be even larger.

### Fractional atomic coordinates and isotropic or equivalent isotropic displacement parameters (Å^2^)

**Table:**

	*x*	*y*	*z*	*U*_iso_*/*U*_eq_	Occ. (<1)
Nd1	0.48488 (2)	0.51184 (2)	0.18894 (2)	0.0276 (1)	
Cl1	0.49345 (10)	0.66402 (8)	−0.04455 (7)	0.0285 (3)	
Cl11	0.79337 (11)	0.51474 (10)	0.06963 (10)	0.0421 (3)	
O11	0.2356 (3)	0.4935 (3)	0.1820 (3)	0.0523 (10)	
O12	0.4711 (4)	0.7381 (3)	0.1875 (3)	0.0550 (12)	
O13	0.2865 (6)	0.5264 (5)	0.3836 (3)	0.111 (2)	
O14	0.5955 (5)	0.5337 (3)	0.3421 (3)	0.0655 (13)	
O15	0.4930 (4)	0.2980 (3)	0.3127 (2)	0.0432 (9)	
N31	−0.00665 (19)	−0.11195 (8)	1.07329 (14)	0.0521 (19)	0.500
N32	0.0280 (4)	0.25416 (14)	0.8530 (3)	0.0521 (19)	0.500
C31	0.00000	0.00000	1.00000	0.0413 (12)	
C32	0.14423 (11)	0.0583 (2)	0.9163 (3)	0.0413 (12)	0.500
C33	0.1501 (3)	0.1728 (2)	0.8432 (3)	0.0413 (12)	0.500
C34	−0.1136 (3)	0.20203 (19)	0.9333 (4)	0.0413 (12)	0.500
C35	−0.12188 (16)	0.08640 (17)	1.0072 (3)	0.0413 (12)	0.500
C36	−0.1501 (3)	−0.1736 (2)	1.1571 (3)	0.098 (5)	0.500
C37	0.1136 (3)	−0.20276 (19)	1.0670 (4)	0.098 (5)	0.500
N11	0.9754 (4)	−0.0990 (4)	0.3994 (4)	0.0544 (14)	
N12	1.0009 (6)	0.2582 (4)	0.1682 (4)	0.0614 (18)	
C11	0.9844 (4)	0.0171 (4)	0.3236 (4)	0.0362 (12)	
C12	1.1192 (5)	0.0674 (5)	0.2318 (4)	0.0488 (16)	
C13	1.1231 (6)	0.1873 (5)	0.1574 (4)	0.0572 (17)	
C14	0.8722 (7)	0.2147 (5)	0.2527 (6)	0.068 (2)	
C15	0.8601 (5)	0.0985 (5)	0.3294 (5)	0.0567 (17)	
C16	0.8358 (7)	−0.1516 (6)	0.4911 (6)	0.083 (2)	
C17	1.1055 (7)	−0.1817 (6)	0.3913 (7)	0.088 (3)	
Cl3	0.51832 (13)	0.21187 (10)	0.57337 (9)	0.0429 (3)	
Cl4	0.07818 (14)	0.49945 (15)	0.64240 (11)	0.0727 (5)	
Cl6	0.50000	0.00000	0.00000	0.0420 (5)	
O1W	0.5008 (5)	0.0727 (3)	0.2546 (3)	0.0672 (13)	
H111	0.14610	0.49370	0.23020	0.0790*	
H112	0.23500	0.49450	0.11120	0.0790*	
H121	0.48380	0.80390	0.12760	0.0820*	
H122	0.46940	0.76020	0.25090	0.0820*	
H131	0.23040	0.55930	0.34170	0.1670*	
H132	0.23300	0.51220	0.45890	0.1670*	
H141	0.55620	0.47610	0.40730	0.0980*	
H142	0.68760	0.51610	0.31140	0.0980*	
H151	0.50200	0.22980	0.28800	0.0650*	
H152	0.50060	0.28000	0.38360	0.0650*	
H32	0.23430	0.01240	0.91520	0.0490*	0.500
H32A	0.03790	0.33510	0.81110	0.0620*	0.500
H33	0.24240	0.20160	0.78040	0.0490*	0.500
H34	−0.20210	0.25030	0.93380	0.0490*	0.500
H35	−0.21530	0.05930	1.06910	0.0490*	0.500
H36A	−0.13100	−0.25800	1.20490	0.1460*	0.500
H36B	−0.21150	−0.18420	1.10930	0.1460*	0.500
H36C	−0.20330	−0.11950	1.21270	0.1460*	0.500
H37A	0.07940	−0.28050	1.13340	0.1460*	0.500
H37B	0.19810	−0.16470	1.07540	0.1460*	0.500
H37C	0.14530	−0.22520	0.98780	0.1460*	0.500
H12	1.00550	0.33520	0.11860	0.0740*	
H12A	1.20780	0.01730	0.22180	0.0590*	
H13	1.21510	0.22070	0.09680	0.0690*	
H14	0.78600	0.26750	0.25900	0.0820*	
H15	0.76560	0.07030	0.38910	0.0680*	
H16A	0.78800	−0.08910	0.53910	0.1240*	
H16B	0.85550	−0.23100	0.54560	0.1240*	
H16C	0.76920	−0.17030	0.45080	0.1240*	
H17A	1.14130	−0.20270	0.31140	0.1320*	
H17B	1.07790	−0.26110	0.45660	0.1320*	
H17C	1.18490	−0.13720	0.40040	0.1320*	
H11W	0.49450	−0.00300	0.30050	0.1000*	
H12W	0.50320	0.06830	0.18430	0.1000*	

### Atomic displacement parameters (Å^2^)

**Table:**

	*U*^11^	*U*^22^	*U*^33^	*U*^12^	*U*^13^	*U*^23^
Nd1	0.0410 (1)	0.0217 (1)	0.0170 (1)	0.0017 (1)	−0.0077 (1)	−0.0030 (1)
Cl1	0.0433 (5)	0.0198 (4)	0.0207 (4)	0.0021 (3)	−0.0107 (3)	−0.0029 (3)
Cl11	0.0362 (5)	0.0397 (5)	0.0472 (6)	−0.0027 (4)	−0.0187 (4)	0.0017 (4)
O11	0.0269 (15)	0.070 (2)	0.0489 (18)	0.0026 (14)	0.0025 (13)	−0.0175 (16)
O12	0.114 (3)	0.0252 (14)	0.0345 (16)	0.0054 (16)	−0.0362 (18)	−0.0082 (12)
O13	0.150 (5)	0.107 (4)	0.0282 (19)	0.053 (3)	0.013 (2)	−0.011 (2)
O14	0.118 (3)	0.0475 (19)	0.0464 (19)	0.008 (2)	−0.049 (2)	−0.0110 (15)
O15	0.075 (2)	0.0263 (14)	0.0281 (14)	−0.0017 (14)	−0.0222 (14)	0.0009 (11)
N31	0.061 (4)	0.033 (3)	0.056 (3)	−0.002 (3)	−0.021 (3)	0.002 (2)
N32	0.061 (4)	0.033 (3)	0.056 (3)	−0.002 (3)	−0.021 (3)	0.002 (2)
C31	0.031 (2)	0.041 (2)	0.047 (2)	0.0040 (17)	−0.0102 (17)	−0.0080 (18)
C32	0.031 (2)	0.041 (2)	0.047 (2)	0.0040 (17)	−0.0102 (17)	−0.0080 (18)
C33	0.031 (2)	0.041 (2)	0.047 (2)	0.0040 (17)	−0.0102 (17)	−0.0080 (18)
C34	0.031 (2)	0.041 (2)	0.047 (2)	0.0040 (17)	−0.0102 (17)	−0.0080 (18)
C35	0.031 (2)	0.041 (2)	0.047 (2)	0.0040 (17)	−0.0102 (17)	−0.0080 (18)
C36	0.091 (8)	0.081 (8)	0.096 (9)	0.004 (6)	−0.028 (7)	0.014 (7)
C37	0.091 (8)	0.081 (8)	0.096 (9)	0.004 (6)	−0.028 (7)	0.014 (7)
N11	0.050 (2)	0.044 (2)	0.060 (3)	−0.0031 (18)	−0.0208 (19)	0.0069 (19)
N12	0.093 (4)	0.035 (2)	0.064 (3)	−0.006 (2)	−0.042 (3)	−0.0022 (19)
C11	0.035 (2)	0.033 (2)	0.037 (2)	−0.0004 (16)	−0.0106 (17)	−0.0046 (17)
C12	0.034 (2)	0.054 (3)	0.051 (3)	−0.001 (2)	−0.008 (2)	−0.009 (2)
C13	0.061 (3)	0.060 (3)	0.043 (3)	−0.027 (3)	−0.009 (2)	−0.003 (2)
C14	0.073 (4)	0.048 (3)	0.080 (4)	0.024 (3)	−0.030 (3)	−0.013 (3)
C15	0.041 (3)	0.056 (3)	0.060 (3)	0.012 (2)	−0.009 (2)	−0.007 (2)
C16	0.074 (4)	0.084 (4)	0.066 (4)	−0.033 (3)	−0.018 (3)	0.024 (3)
C17	0.081 (4)	0.056 (4)	0.117 (6)	0.021 (3)	−0.047 (4)	0.008 (4)
Cl3	0.0650 (7)	0.0375 (5)	0.0298 (5)	0.0030 (5)	−0.0210 (5)	−0.0083 (4)
Cl4	0.0507 (7)	0.0898 (10)	0.0383 (6)	0.0214 (7)	0.0100 (5)	0.0094 (6)
Cl6	0.0588 (9)	0.0270 (7)	0.0404 (8)	−0.0014 (6)	−0.0198 (7)	−0.0040 (6)
O1W	0.128 (3)	0.0297 (16)	0.050 (2)	0.0021 (19)	−0.042 (2)	−0.0041 (14)

### Geometric parameters (Å, º)

**Table:**

Nd1—Cl1	2.8298 (8)	N12—H12	0.8800
Nd1—Cl11	2.7852 (11)	C31—C35^ii^	1.417 (2)
Nd1—O11	2.429 (3)	C31—C32^ii^	1.467 (2)
Nd1—O12	2.426 (3)	C31—C35	1.417 (2)
Nd1—O13	2.477 (4)	C31—C32	1.467 (2)
Nd1—O14	2.479 (4)	C32—C33	1.306 (4)
Nd1—O15	2.404 (3)	C34—C35	1.318 (4)
Nd1—Cl1^i^	2.8345 (9)	C32—H32	0.9500
O11—H111	0.8500	C33—H33	0.9500
O11—H112	0.8500	C34—H34	0.9500
O12—H121	0.8500	C35—H35	0.9500
O12—H122	0.8500	C36—H36B	0.9800
O13—H131	0.8500	C36—H36C	0.9800
O13—H132	0.8500	C36—H36A	0.9800
O14—H141	0.8500	C37—H37A	0.9800
O14—H142	0.8500	C37—H37B	0.9800
O15—H151	0.8500	C37—H37C	0.9800
O15—H152	0.8500	C11—C15	1.404 (7)
N31—C31	1.2855 (12)	C11—C12	1.408 (6)
N31—C36	1.474 (4)	C12—C13	1.360 (7)
N31—C37	1.435 (3)	C14—C15	1.333 (8)
N32—C33	1.385 (4)	C12—H12A	0.9500
N32—C34	1.418 (5)	C13—H13	0.9500
O1W—H12W	0.8500	C14—H14	0.9500
O1W—H11W	0.8500	C15—H15	0.9500
N32—H32A	0.8800	C16—H16B	0.9800
N11—C11	1.330 (6)	C16—H16C	0.9800
N11—C17	1.452 (8)	C16—H16A	0.9800
N11—C16	1.446 (8)	C17—H17C	0.9800
N12—C13	1.322 (8)	C17—H17A	0.9800
N12—C14	1.319 (9)	C17—H17B	0.9800
			
Cl1—Nd1—Cl11	81.28 (3)	C32—C31—C35^ii^	66.36 (14)
Cl1—Nd1—O11	74.96 (8)	C32—C31—C32^ii^	180.00
Cl1—Nd1—O12	69.82 (8)	C35—C31—C35^ii^	180.00
Cl1—Nd1—O13	124.05 (12)	C32^ii^—C31—C35^ii^	113.64 (14)
Cl1—Nd1—O14	134.06 (8)	N31^ii^—C31—C32	58.94 (12)
Cl1—Nd1—O15	146.10 (7)	N31—C31—C35^ii^	55.70 (14)
Cl1—Nd1—Cl1^i^	74.27 (2)	C32^ii^—C31—C35	66.36 (14)
Cl11—Nd1—O11	148.14 (8)	C32—C31—C35	113.64 (14)
Cl11—Nd1—O12	94.54 (9)	N31^ii^—C31—C35^ii^	124.30 (14)
Cl11—Nd1—O13	144.01 (13)	N31^ii^—C31—C32^ii^	121.06 (12)
Cl11—Nd1—O14	74.88 (10)	C31—C32—C33	120.66 (19)
Cl11—Nd1—O15	91.79 (9)	N32—C33—C32	123.0 (3)
Cl1^i^—Nd1—Cl11	79.91 (3)	N32—C34—C35	119.7 (3)
O11—Nd1—O12	96.80 (12)	C31—C35—C34	124.0 (3)
O11—Nd1—O13	67.84 (15)	C33—C32—H32	120.00
O11—Nd1—O14	136.94 (12)	C31—C32—H32	120.00
O11—Nd1—O15	96.19 (12)	C32—C33—H33	119.00
Cl1^i^—Nd1—O11	73.49 (8)	N32—C33—H33	118.00
O12—Nd1—O13	74.71 (15)	N32—C34—H34	120.00
O12—Nd1—O14	73.57 (12)	C35—C34—H34	120.00
O12—Nd1—O15	144.06 (10)	C31—C35—H35	118.00
Cl1^i^—Nd1—O12	144.08 (8)	C34—C35—H35	118.00
O13—Nd1—O14	69.15 (16)	H36A—C36—H36B	109.00
O13—Nd1—O15	79.52 (14)	H36A—C36—H36C	109.00
Cl1^i^—Nd1—O13	128.31 (13)	N31—C36—H36C	109.00
O14—Nd1—O15	74.08 (11)	N31—C36—H36A	110.00
Cl1^i^—Nd1—O14	136.47 (9)	H36B—C36—H36C	109.00
Cl1^i^—Nd1—O15	71.85 (7)	N31—C36—H36B	109.00
Nd1—Cl1—Nd1^i^	105.74 (3)	H37B—C37—H37C	109.00
Nd1—O11—H111	137.00	H37A—C37—H37C	109.00
Nd1—O11—H112	113.00	N31—C37—H37B	110.00
H111—O11—H112	109.00	N31—C37—H37A	109.00
Nd1—O12—H121	130.00	H37A—C37—H37B	110.00
Nd1—O12—H122	119.00	N31—C37—H37C	109.00
H121—O12—H122	110.00	C12—C11—C15	115.1 (4)
Nd1—O13—H131	88.00	N11—C11—C15	122.4 (4)
Nd1—O13—H132	163.00	N11—C11—C12	122.5 (4)
H131—O13—H132	108.00	C11—C12—C13	120.3 (5)
Nd1—O14—H141	107.00	N12—C13—C12	121.1 (5)
Nd1—O14—H142	104.00	N12—C14—C15	121.7 (6)
H141—O14—H142	109.00	C11—C15—C14	121.2 (5)
Nd1—O15—H151	125.00	C11—C12—H12A	120.00
Nd1—O15—H152	125.00	C13—C12—H12A	120.00
H151—O15—H152	109.00	C12—C13—H13	119.00
C31—N31—C36	122.21 (17)	N12—C13—H13	119.00
C31—N31—C37	125.7 (2)	N12—C14—H14	119.00
C36—N31—C37	110.75 (19)	C15—C14—H14	119.00
C33—N32—C34	117.4 (2)	C14—C15—H15	119.00
H11W—O1W—H12W	109.00	C11—C15—H15	119.00
C34—N32—H32A	121.00	N11—C16—H16A	109.00
C33—N32—H32A	121.00	N11—C16—H16B	109.00
C11—N11—C16	122.2 (4)	H16A—C16—H16B	109.00
C11—N11—C17	121.4 (5)	H16A—C16—H16C	109.00
C16—N11—C17	116.4 (5)	N11—C16—H16C	109.00
C13—N12—C14	120.6 (5)	H16B—C16—H16C	109.00
C13—N12—H12	120.00	N11—C17—H17B	109.00
C14—N12—H12	120.00	N11—C17—H17C	109.00
N31—C31—C35	124.30 (14)	N11—C17—H17A	109.00
N31—C31—C32	121.06 (12)	H17A—C17—H17C	109.00
N31—C31—N31^ii^	180.00	H17B—C17—H17C	110.00
N31^ii^—C31—C35	55.70 (14)	H17A—C17—H17B	109.00
N31—C31—C32^ii^	58.94 (12)		
			
Cl11—Nd1—Cl1—Nd1^i^	81.90 (4)	C33—N32—C34—C35	9.3 (5)
O11—Nd1—Cl1—Nd1^i^	−76.70 (9)	C16—N11—C11—C12	−178.2 (5)
O12—Nd1—Cl1—Nd1^i^	−179.96 (10)	C16—N11—C11—C15	1.9 (7)
O13—Nd1—Cl1—Nd1^i^	−125.94 (16)	C17—N11—C11—C12	−0.3 (7)
O14—Nd1—Cl1—Nd1^i^	140.86 (13)	C17—N11—C11—C15	179.8 (5)
O15—Nd1—Cl1—Nd1^i^	1.78 (17)	C14—N12—C13—C12	0.8 (8)
Cl1^i^—Nd1—Cl1—Nd1^i^	0.00 (4)	C13—N12—C14—C15	−0.3 (9)
Cl1—Nd1—Cl1^i^—Nd1^i^	0.00 (3)	N31^ii^—C31—C32—C33	0.9 (2)
Cl11—Nd1—Cl1^i^—Nd1^i^	−83.69 (4)	C35—C31—C32—C33	−10.1 (3)
O11—Nd1—Cl1^i^—Nd1^i^	78.59 (9)	N31—C31—C32—C33	−179.1 (2)
O12—Nd1—Cl1^i^—Nd1^i^	0.06 (16)	C32—C31—C35—C34	10.0 (3)
O13—Nd1—Cl1^i^—Nd1^i^	121.24 (15)	C35^ii^—C31—C32—C33	169.9 (3)
O14—Nd1—Cl1^i^—Nd1^i^	−138.80 (13)	N31—C31—C35—C34	178.6 (2)
O15—Nd1—Cl1^i^—Nd1^i^	−178.95 (10)	N31^ii^—C31—C35—C34	−1.4 (2)
C36—N31—C31—C32	−179.1 (2)	C32^ii^—C31—C35—C34	−170.0 (3)
C36—N31—C31—C35	13.0 (2)	C31—C32—C33—N32	11.0 (4)
C36—N31—C31—C32^ii^	0.88 (19)	N32—C34—C35—C31	−10.1 (5)
C36—N31—C31—C35^ii^	−167.0 (2)	N11—C11—C12—C13	−179.2 (5)
C37—N31—C31—C32	−13.5 (3)	C15—C11—C12—C13	0.7 (7)
C37—N31—C31—C35	178.6 (2)	N11—C11—C15—C14	179.7 (5)
C37—N31—C31—C32^ii^	166.5 (3)	C12—C11—C15—C14	−0.3 (8)
C37—N31—C31—C35^ii^	−1.4 (2)	C11—C12—C13—N12	−1.0 (8)
C34—N32—C33—C32	−10.2 (5)	N12—C14—C15—C11	0.1 (9)

Symmetry codes: (i) −*x*+1, −*y*+1, −*z*; (ii) −*x*, −*y*, −*z*+2.

### Hydrogen-bond geometry (Å, º)

**Table:**

*D*—H···*A*	*D*—H	H···*A*	*D*···*A*	*D*—H···*A*
O11—H111···Cl4^iii^	0.85	2.16	3.010 (3)	177
O11—H112···Cl11^i^	0.85	2.30	3.149 (4)	173
O12—H121···Cl6^iv^	0.85	2.24	3.080 (3)	168
O12—H122···Cl3^v^	0.85	2.25	3.089 (3)	171
O13—H131···O11	0.85	2.19	2.738 (6)	122
O13—H131···Cl4^iii^	0.85	2.99	3.631 (6)	134
O13—H132···Cl4	0.85	2.16	3.005 (4)	172
O14—H142···Cl4^v^	0.85	2.46	3.159 (4)	140
O14—H142···Cl11	0.85	2.73	3.210 (4)	118
O15—H151···O1*W*	0.85	1.84	2.683 (4)	172
O15—H152···Cl3	0.85	2.26	3.109 (3)	175
N12—H12···Cl11^vi^	0.88	2.65	3.358 (5)	138
N12—H12···Cl11	0.88	2.78	3.451 (5)	134
O1*W*—H11*W*···Cl3^vii^	0.85	2.35	3.197 (3)	179
O1*W*—H12*W*···Cl6	0.85	2.52	3.349 (3)	167
N32—H32*A*···Cl4	0.88	2.26	3.0808 (16)	156
C34—H34···Cl1^iii^	0.95	2.82	3.703 (3)	155

Symmetry codes: (i) −*x*+1, −*y*+1, −*z*; (iii) −*x*, −*y*+1, −*z*+1; (iv) *x*, *y*+1, *z*; (v) −*x*+1, −*y*+1, −*z*+1; (vi) −*x*+2, −*y*+1, −*z*; (vii) −*x*+1, −*y*, −*z*+1.

## References

[bb1] Agilent (2011). *CrysAlis PRO* Agilent Technologies Ltd, Yarnton, England.

[bb2] Altomare, A., Cascarano, G., Giacovazzo, C. & Guagliardi, A. (1993). *J. Appl. Cryst.* **26**, 343–350.

[bb3] Benslimane, M., Merazig, H., Daran, J.-C. & Zeghouan, O. (2012). *Acta Cryst* E**68**, m1321–m1322. 10.1107/S1600536812040901 PMC3515091 23284318

[bb4] Bernstein, J., Davis, R. E., Shimoni, L. & Chang, N.-L. (1995). *Angew. Chem. Int. Ed. Engl.* **34**, 1555–1573.

[bb5] Burnett, M. N. & Johnson, C. K. (1996). *ORTEPIII* Report ORNL-6895. Oak Ridge National Laboratory, Tennessee, USA.

[bb6] Chao, M., Schempp, E. & Rosenstein, D. (1977). *Acta Cryst.* B**33**, 1820–1823.

[bb7] Farrugia, L. J. (2012). *J. Appl. Cryst.* **45**, 849–854.

[bb8] Koon, Y. C., Lo, K. M. & Ng, S. W. (2009). *Acta Cryst.* E**65**, m663. 10.1107/S1600536809017590 PMC2969743 21583025

[bb9] Lo, K. M. & Ng, S. W. (2008). *Acta Cryst.* E**64**, m800. 10.1107/S1600536808013561 PMC2961524 21202487

[bb10] Lo, K. M. & Ng, S. W. (2009). *Acta Cryst.* E**65**, m13. 10.1107/S1600536808040312 PMC2967864 21581489

[bb11] Mayr-Stein, R. & Bolte, M. (2000). *Acta Cryst.* C**56**, e19–e20. 10.1107/S0108270100001888 10851636

[bb12] Sheldrick, G. M. (2008). *Acta Cryst.* A**64**, 112–122. 10.1107/S0108767307043930 18156677

